# Pharmacogenomics: From Basic Research to Clinical Implementation

**DOI:** 10.3390/jpm11080800

**Published:** 2021-08-17

**Authors:** Laura B. Scheinfeldt

**Affiliations:** Coriell Institute for Medical Research, Camden, NJ 08003, USA; lscheinfeldt@coriell.org

The established contribution of genetic variation to drug response has the potential to improve drug efficacy and reduce drug toxicity [1]. The uptake of pharmacogenomics (PGx) in clinical care, however, has been relatively slow despite the documentation and validation of many known genetic determinants of drug response. This special issue, entitled “Pharmacogenomics: From Basic Research to Clinical Implementation,” focuses on the current state of pharmacogenomics and the extensive translational process required for clinical implementation, including the characterization of functionally important PGx variation, the clinical interpretation of PGx variation, clinical PGx decision support, and the incorporation of PGx into clinical care.

Four of the special issue articles, Han et al. [2], Lee et al. [3], Scheinfeldt et al. [1], and Kim et al. [4] focus on the identification, characterization, and documentation of functionally important PGx variation. Kim et al. [4] conducted a longitudinal review of FDA-approved PGx drugs and FDA PGx drug labels. The authors identified a notable increase in PGx content between 2000 and 2020 but also note that the majority of these involved cancer treatment drug labels. This analysis demonstrates the need for more PGx support in non-cancer therapeutics. Han et al. [2] focused on the identification of pharmacogenetic single nucleotide polymorphisms (SNPs) and copy number variation (CNV) in the Korean Genome and Epidemiology Study, which included genome-wide SNP data collected from over 70,000 Korean Genome and Epidemiology Study participants and CNV data collected from 1000 study participants. The authors used their cohort data to confirm the clinical implications of important variants in several pharmacogenes, including *VKORC1*, *CYP2D6*, *CYP2C19*, and *TPMT*. Lee et al. [3] focused on the impact PGx variation at *CYP3A5* has on chronic kidney disease progression. This example demonstrates that PGx variation may impact disease treatment through drug response as well as through physiological effects in kidney that may exacerbate kidney disease. Scheinfeldt et al. [1] took a complementary in silico approach that leveraged the evolutionary history of the genes involved in drug response to predict functionally important pharmacovariants. Not only did they identify over 2000 new putative pharmacovariants, but they demonstrated that these pharmacovariants are common across worldwide communities.

Two of the special issue articles, Silva et al. [5] and Schmidlen et al. [6], focused on PGx clinical decision support. Silva et al. [5] leveraged a Clinical Semantic Network framework to apply a pharmacogenomic model to patient electronic medical record data. They validated this framework with a virtual case study and demonstrated that their approach can identify clinically significant drug–drug and drug–gene interactions. Given the increasingly complex challenges to integrating medical informatics data for clinical decision-making, this framework and others like it will be needed to support precision medication management. Schmidlen et al. [6] conducted a retrospective qualitative analysis of genetic counseling requests from participants in a personalized medicine research study and demonstrated the critical role that genetic counselors play in supporting providers in their communication of PGx results to patients and supporting patients in their understanding of PGx results.

Several of the studies included in this special issue focus more directly on the incorporation of PGx into clinical care. Gill et al. [7] focused on PGx implementation in pediatric care. Importantly, the authors described a framework in which PGx testing is incorporated into the EHR system being used by clinicians involved in the study (in this case, EPIC). Breaux et al. [8] presented an example of PGx implementation for mental health medications involving a collaborative framework of pharmacists and clinicians. The authors found that PGx-led medication changes added minimal short-term cost to patient care and emphasized potential long-term benefits, including improved dosing and reduced adverse drug reactions. Pasternak et al. [9] conducted a retrospective review of medical records and provided a detailed assessment of documented PGx testing. These authors used this review to develop several recommendations for improving clinical PGx testing, including the establishment of a clinical PGx consult service involving pharmacists and clinicians and the application of standardized CPIC terminology. Lanting et al. [10] also focused on challenges to clinical PGx implementation in a complementary prospective manner involving patients, physicians, and pharmacists. While patient and clinician attitudes toward PGx testing were typically positive, the authors documented a need for additional PGx education for clinicians and a clear determination of which clinicians should take primary responsibility for clinical PGx testing. 

Taken together, this body of work builds upon the extensive information already known about contributions of genetic variation to drug response and describes important gaps in this knowledge as well as challenges to the clinical implementation of pharmacogenomics that remain to be addressed. Several examples of clinical PGx implementation in a variety of settings highlight areas of ongoing improvement and momentum toward more broad integration of PGx testing.

## Data Availability

Not applicable.
